# Endoscopy in a Pediatric Patient After Necrotizing Enterocolitis

**DOI:** 10.1097/PG9.0000000000000306

**Published:** 2023-04-18

**Authors:** Erica Chang, Dorothy Rowe, Ashish Patel, Brad Pasternak

**Affiliations:** From the * Division of Pediatric Gastroenterology, Department of Pediatrics, Phoenix Children’s Hospital, Phoenix, AZ; † Department of Pediatric Surgery, Phoenix Children’s Hospital, Phoenix, AZ.

**Keywords:** endoscopy, perforation, pneumatosis

## Abstract

Necrotizing enterocolitis (NEC) is a gastrointestinal condition usually found in premature neonates. Our case involves a full-term, 3-month-old male who was found to have pneumatosis after surgical repair of congenital cardiac defects. After cessation of enteral feeds, nasogastric tube decompression, and broad-spectrum antibiotics, breast milk was reintroduced 8 days after his procedure. Hematochezia developed, but repeat abdominal X-rays were normal with benign abdominal examinations, stable vital signs, and improved laboratory findings. Although feeds were slowly restarted with an amino acid-based formula, hematochezia persisted. Meckel’s scan was negative, and Computerized Tomography revealed diffuse bowel inflammation. Esophagogastroduodenoscopy and flexible sigmoidoscopy were performed for further evaluation which showed stricture and ulceration at the descending colon. This procedure was complicated by perforation with subsequent resection of this segment and diverting ileostomy. Due to the risk of complications, it is suggested to wait at least 6 weeks from acute events such as NEC before performing an endoscopy.

## INTRODUCTION

Necrotizing enterocolitis (NEC) is a life-threatening condition that typically affects premature infants. It is thought that the bowel wall is invaded by bacteria, leading to local inflammation and infection that can subsequently lead to ischemia and perforation. Common clinical features include feeding intolerance, abdominal distension, and hematochezia. Physical examination may reveal tenderness to palpation, abdominal mass, and erythema of the abdominal wall (1). Abdominal X-rays are essential for diagnosing and following the progression of NEC, revealing pneumatosis intestinalis, portal venous air, or dilated loops of the bowel (1). Laboratory tests have limited utility, but leukopenia, hyponatremia, and low serum bicarbonate may also support the diagnosis. Treatment involves supportive care with bowel rest, total parenteral nutrition, and gastric decompression via nasogastric (NG) tube. Broad-spectrum antibiotics are also part of routine therapy. Enteral feeds may be resumed once patients show clinical improvement which may take days to weeks. However, surgical intervention is necessary if the infant does not respond to medical treatment or develops a bowel perforation. Patients may also develop complications such as stricture, short bowel syndrome, and intestinal failure. Currently, the safe timing of endoscopy in patients following NEC is unclear.

## CASE REPORT

This male patient was born full-term via repeat C-section with no prenatal or perinatal complications. At his 2-month well child check, a murmur was noted which led to the discovery of ventricular septal defects, coarctation of the aorta, bicuspid aortic valve, and patent ductus arteriosus. At this time, he was exclusively breastfed and growing well (90%ile for height, 50%ile for weight) without significant medical concerns. At 3 months, he was admitted for coarctation of the aorta and ventricular septal defects repair which proceeded without any major surgical events. However, on postoperative day (POD) 1, he required transfusion for hemoglobin 7.4 g/dL and hematocrit 21.7 with subsequent improvement. A NG tube was placed to administer aspirin, and postplacement kidney, ureter, and bladder X-ray incidentally revealed pneumatosis in the right upper quadrant. No prior images were available for comparison. Modified Bell stage was IIA based on radiographic signs, and pediatric surgery was consulted. The patient was made nil per os (NPO) and started on Zosyn until POD 8. Feeds were then restarted with breastmilk; however, the patient had two large bloody stools and was made NPO again. Aspirin was held due to hematochezia. C-Reactive Protein was noted to be 2.6 mg/dL (normal <0.9 mg/dL) on POD 12. Repeat kidney, ureter, and bladder X-ray on POD 12 and 13 were normal. The gastroenterology team was consulted on POD 13, at which time the patient’s examination revealed a slight murmur but overall unremarkable with a soft, non-tender, nondistended abdomen. Labs on POD 13 revealed down-trending leukocytes (12.8 × 10^3^/uL), up-trending platelets (754,000/uL), and increasing hemoglobin (12.5 g/dL).

**FIGURE 1.**
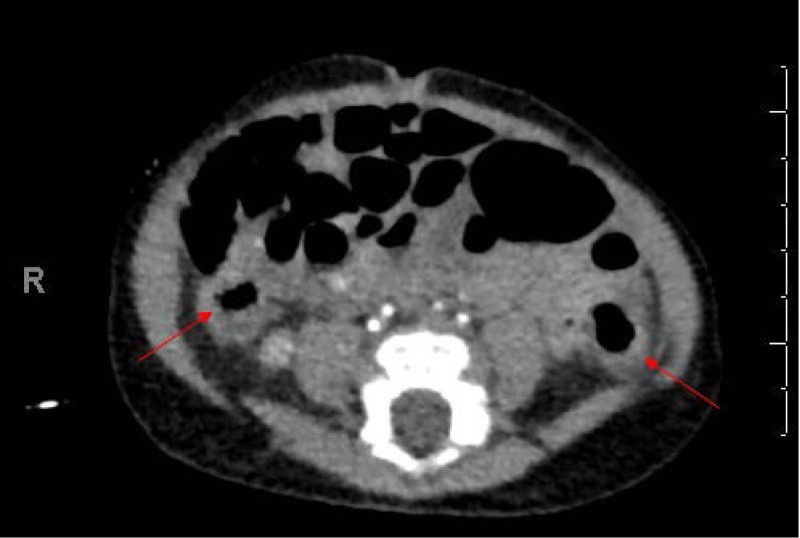
Hyperenhancement of the ascending and descending colon mucosal lining suggestive of inflammation or infection.

On POD 14, feeds were restarted with Elecare due to concern for cow’s milk protein intolerance (CMPI). Hematochezia was subsequently noted, and hemoglobin decreased to 9.3 g/dL. International Normalized Ratio was 1.2, Prothrombin Time 14.7 sec, and Activated Partial Thromboplastin Clotting Time 29.2 sec. He was made NPO again, and pantoprazole was started. At this time, the patient continued to be well appearing with benign examinations (Modified Bell stage IB). Consideration was given to possible Meckel’s diverticulum, gastric ulcer, or arteriovenous malformation (AVM) which would likely require embolization or surgical intervention. On POD 15, Meckel’s scan was negative. Computed tomography angiography revealed findings suggestive of diffuse bowel inflammation or infection with multiple segments of the small bowel and colon that demonstrated hyperenhancement of the mucosal lining (Fig. 1). However, the etiology of inflammation was unknown, and AVM was not definitively ruled out.

**FIGURE 2.**
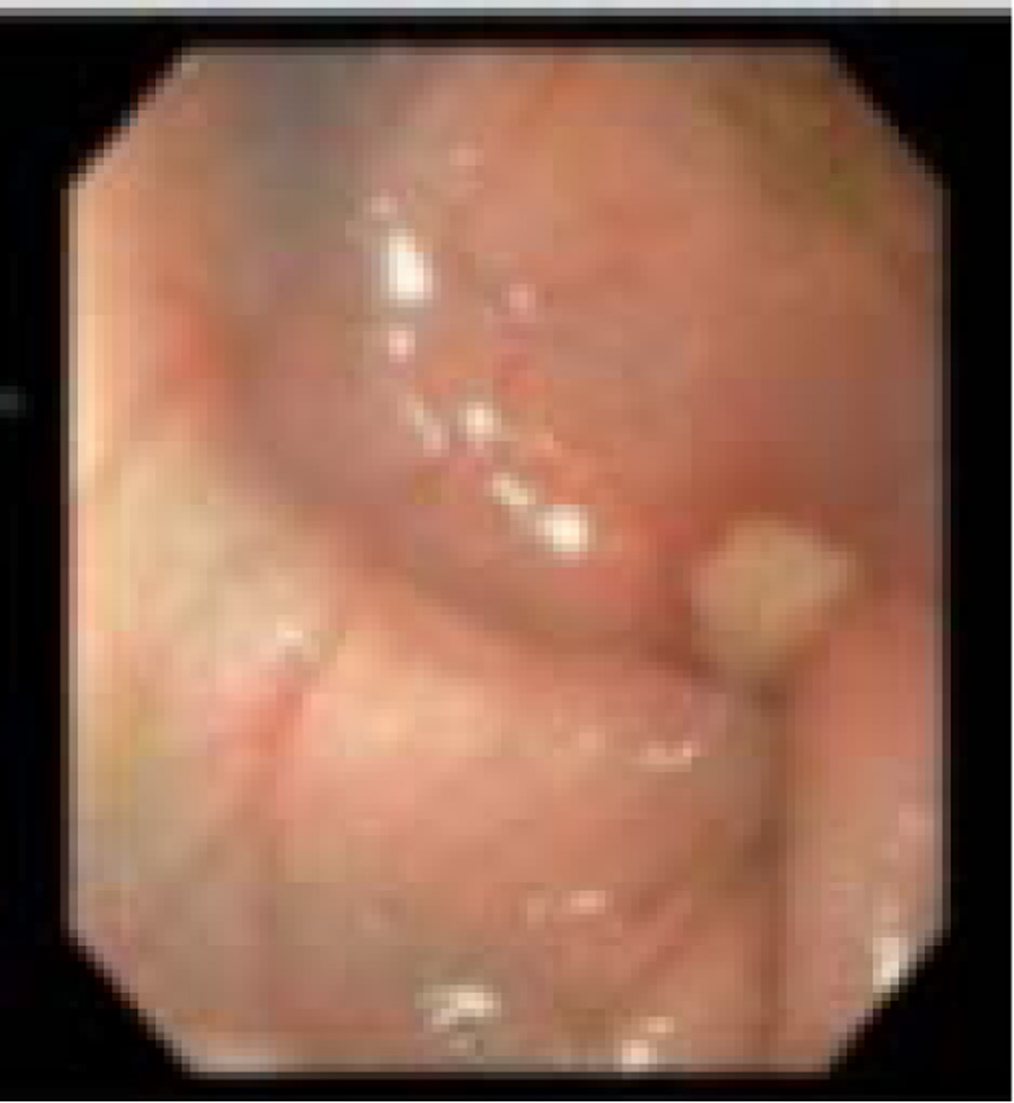
Sigmoidoscopy revealed a single ulceration at the descending colon.

Esophagogastroduodenoscopy (EGD) and flexible sigmoidoscopy were performed on POD 16 to assess for AVM versus other etiologies such as very early onset inflammatory bowel disease or allergic colitis. EGD was unremarkable, but flexible sigmoidoscopy revealed a small ulcer in the descending colon (Fig. 2) and was complicated by a perforation. The surgeons performed an emergent diverting ileostomy. A segment of descending colon approximately 12 cm long (starting 6 cm proximal to 6 cm distal to the perforation) was resected, and a stricture was identified within this segment. Pathology reports indicated hemorrhagic ischemia with perforation and serositis.

Breast milk was eventually restarted on POD 19, and the patient was discharged after the resolution of postoperative ileus. Upon outpatient follow-up, he had no feeding difficulties and good ostomy output. Contrast enema was normal, and ileostomy takedown was successfully performed on POD 112 without complications.

## DISCUSSION

Although NEC predominantly occurs in preterm infants, it can also be observed in term infants with congenital heart disease such as our patient (2). This is likely due to a low perfusion state in the perioperative period leading to ischemia (3). If NEC develops, early refeeding is encouraged due to a lower risk of recurrent NEC and/or post-NEC stricture (4). However, further workup is warranted if hematochezia continues despite appropriate management. Interestingly, our patient had ongoing, painless hematochezia in the setting of normal serial abdominal X-rays, benign examinations, and no coagulopathy. In such cases, differentials may include repeat NEC, CMPI, Meckel’s diverticulum, AVM, very early onset inflammatory bowel disease, and less likely gastric ulcer/bleeding with brisk transit. If Meckel’s scan is negative and bleeding persists despite a trial of cow’s milk elimination, further imaging such as computed tomography angiography may be pursued to assess for AVM. If AVM cannot be ruled out definitively and bleeding persists despite initial management, EGD/flexible sigmoidoscopy may be considered. In our case, endoscopy revealed a single ulceration of the descending colon which was likely due to NEC history. These findings were not identified on imaging which is consistent with previous studies which showed that radiographic signs for NEC have high specificity but low sensitivity (5). This patient also had intestinal stricture which may start to form during the acute phase of the disease but have no obvious abnormalities on X-ray (6). Similarly, other studies evaluating NEC patients via flexible proctosigmoidoscopy and colonic mucosal biopsy revealed colitis of varying severity from mild erythema to severe ulceration and spontaneous hemorrhage; active bleeding from ulcers at the base of strictures was also identified (7). While endoscopy can help differentiate strictures and other bleeding causes such as AVM or CMPI to guide management, there are currently no studies in literature discussing the safe timing of scopes after NEC. In general, endoscopy should be delayed if possible until active inflammation subsides due to the increased risk of perforation (8). During this period, it may be useful to pursue alternative modalities such as doppler ultrasonography which may provide additional information beyond radiographs regarding bowel wall thickness, echogenicity, perfusion, or other findings indicative of NEC. However, a meta-analysis revealed that bowel ultrasound findings also have low sensitivity and high specificity (9). Thus, flexible sigmoidoscopy or colonoscopy may be warranted if hematochezia persists and the etiology is still unclear. In such a case, it is suggested to wait at least 6 weeks from acute events (i.e. NEC) before performing endoscopy, as the risk of significant complications if done before this rarely outweighs potential benefits to the patient (8).

## ACKNOWLEDGMENT

Informed patient consent was obtained for publication of the case details.
